# Multi-phasic bi-directional chemotactic responses of the growth cone

**DOI:** 10.1038/srep36256

**Published:** 2016-11-03

**Authors:** Honda Naoki, Makoto Nishiyama, Kazunobu Togashi, Yasunobu Igarashi, Kyonsoo Hong, Shin Ishii

**Affiliations:** 1 Graduate School of Medicine, Kyoto University, Sakyo, Kyoto, Japan; 2 Imaging Platform for Spatio-temporal Information, Kyoto University, Sakyo, Kyoto, Japan; 3 Department of Biochemistry, New York University School of Medicine, New York, USA; 4 Kasah Technology Inc. New York, New York, USA; 5 Olympus Software Technology Corporation, Hachioji, Tokyo, Japan; 6 Graduate School of Informatics, Kyoto University, Sakyo, Kyoto, Japan

## Abstract

The nerve growth cone is bi-directionally attracted and repelled by the same cue molecules depending on the situations, while other non-neural chemotactic cells usually show uni-directional attraction or repulsion toward their specific cue molecules. However, how the growth cone differs from other non-neural cells remains unclear. Toward this question, we developed a theory for describing chemotactic response based on a mathematical model of intracellular signaling of activator and inhibitor. Our theory was first able to clarify the conditions of attraction and repulsion, which are determined by balance between activator and inhibitor, and the conditions of uni- and bi-directional responses, which are determined by dose-response profiles of activator and inhibitor to the guidance cue. With biologically realistic sigmoidal dose-responses, our model predicted tri-phasic turning response depending on intracellular Ca^2+^ level, which was then experimentally confirmed by growth cone turning assays and Ca^2+^ imaging. Furthermore, we took a reverse-engineering analysis to identify balanced regulation between CaMKII (activator) and PP1 (inhibitor) and then the model performance was validated by reproducing turning assays with inhibitions of CaMKII and PP1. Thus, our study implies that the balance between activator and inhibitor underlies the multi-phasic bi-directional turning response of the growth cone.

During development, the connectivity of neural circuits is determined by axon guidance, a chemotactic process in which the axonal growth cone orients its migrating direction in response to extracellular guidance cues^1^. The motile growth cone, unlike other chemotactic cells, has the unique character of being attracted or repelled by the same guidance cue depending on its biological environment (this character denotes bi-directionality hereafter)^2^. These chemo-attraction and chemo-repulsion responses are dynamically regulated to achieve a mature, functional nervous system^3^,^4^. The elucidation of the molecular mechanisms by which bi-directional attractive and repulsive responses of the growth cone are regulated is key for understanding circuit formation in the developing nervous system.

Non-neural cells, such as *Dictyostelium discoideum* and immune cells, are persistently attracted or repelled by specific cue molecules (this character denotes uni-directionality hereafter). This uni-directional chemotaxis is correlated with the polarized accumulation of an intracellular signaling molecule, *i.e.*, a gradient of phosphoinositol-3,4,5-trisphosphate (PIP3)^5^. Such polarized accumulation of PIP3 was generated by a local excitation-global inhibition (LEGI) model, in which the activator and inhibitor locally and dispersedly function, respectively^6^,^7^,^8^,^9^. It has been thought that activator and inhibitor could be phosphoinositide 3-kinase (PI3K) and the lipid phosphatase, phosphatase and tensin homolog (PTEN), each of which synthesizes PIP3 from phosphoinositol-4,5-bisphosphate (PIP2) and reciprocally metabolizes PIP3 to PIP2, respectively. Note that LEGI mechanism could also be implemented by other pathways, *e.g*., the small GTPase Ras^8^,^10^,^11^, because the cells in which all PI3Ks are knocked out still display reasonable chemotaxis with slower migration speed^12^. In the LEGI model, however, how a cell senses the gradient of chemorepellents has not been fully addressed.

In growth cones, the intracellular levels of second messengers, cyclic nucleotides (cAMP and cGMP) and calcium ion (Ca^2+^), are known to regulate bi-directional turning in response to the same guidance cue. For example, Netrin-1 leads to the production of cAMP and cGMP, which activate cAMP-dependent protein kinase (PKA) and cGMP-dependent protein kinase (PKG), respectively, and, in turn, induces Ca^2+^ influx^13^ (Fig. 1A,B). Extracellular gradients of diffusible guidance signals are translated into intracellular Ca^2+^ gradients in the same direction as the extracellular gradients, regardless of attraction or repulsion (Fig. 1C)^13^,^14^,^15^,^16^. A gradient of Netrin-1 induces attraction by increasing both the ratio of cAMP to cGMP and the Ca^2+^ level in the growth cone. However, if the ratio of cAMP to cGMP or the level of Ca^2+^ decreases, then the same Netrin-1 gradient induces repulsion^13^,^14^. Moreover, Ca^2+^ imaging studies have demonstrated that high and low increases in intracellular Ca^2+^ gradients are generated during attraction and repulsion, respectively^15^. The basal (resting) level of intracellular Ca^2+^ has also been shown to modulate the growth cone turning direction: a high basal level of Ca^2+^ results in attraction, whereas a low basal level of Ca^2+^ results in repulsion, given the same increase in localized Ca^2+^ 17,18. Thus, the intracellular Ca^2+^ level is a key mediator that governs the bi-directional turning response of the growth cone (Fig. 1C).

Calmodulin (CaM)-dependent kinase II (CaMKII) and Protein phosphatase 1 (PP1) function in the growth cone as an activator-inhibitor system in the downstream of Ca^2+^, similarly as PI3K and PTEN function in non-neural cells; CaMKII and PP1 act as an activator and inhibitor, respectively, of the effectors that regulate growth cone motility, *e.g.*, Rac1 and Cdc42 (Fig. 1B)^19^,^20^,^21^,^22^. Importantly, these downstream molecules also modulate the turning direction in response to an external cue: CaMKII triggers attraction, whereas PP1 triggers repulsion^18^. Thus, the downstream Ca^2+^ signaling pathway ultimately decodes the external signal to induce either attractive or repulsive growth cone behavior. How, then, does the activator-inhibitor system regulate the bi-directional turning behaviors of the growth cone in response to the same external cue?

Recently, two computational models have been proposed with interests in chemotactic response of growth cones. Forbes *et al.* proposed a model of growth cone Ca^2+^ signaling^23^, by extending the synaptic plasticity model incorporating CaMKII bi-stability^24^. On the other hand, Roccasalvo *et al.* developed a reaction-diffusion model of self-enhancement dynamics of Ca^2+^ in two-dimensional growth cone^25^. Although these models successfully reproduced bi-directional turning behaviors of growth cones, essential difference in the underlying mechanism between uni-directional chemotactic cells and bi-directional growth cones has been largely unknown.

Here, we proposed a mathematical model to generally address both uni- and bi-directional chemotactic responses, based on an activator-inhibitor system shared by many chemotactic cells. We then established a general theory that describes the mechanistic difference between non-neural chemotactic cells showing uni-directionality and growth cones showing bi-directionality. Based on the model analysis, we theoretically predicted that the turning response of the growth cone could multi-phasically change, *e.g*., from repulsion, attraction to repulsion, as intracellular Ca^2+^ increased, and experimentally validated the prediction using growth cone turning assays and Ca^2+^ imaging. Furthermore, we reverse engineered the model parameters to fit the growth cone turning assays, so that its predictive performance was experimentally validated by pharmacological suppression of the activator (CaMKII) or inhibitor (PP1).

## Results

### A mathematical model of activator-inhibitor system

**Figure 1:**
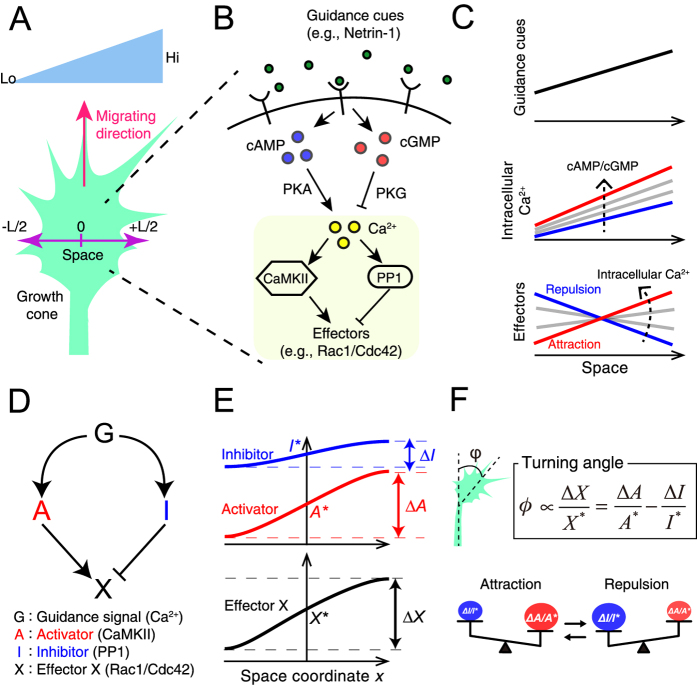
The mathematical growth cone model encompasses an activator and inhibitor system. (**A**) The schematic drawing depicting the one-dimensional model growth cone encountering an extracellular gradient of guidance cues. (**B**) In the growth cone, guidance cues, *e.g*., Netrin-1, lead to the production of cAMP and cGMP, which activate PKA and PKG, respectively, in turn inducing a Ca^2+^ increase. The intracellular Ca^2+^ up-regulates CaMKII and PP1, which function as an activator and inhibitor, respectively, of their effector to regulate growth cone motility (referred to as ‘X’ in the text and (**D**)), *e.g*., Rac1 and Cdc42. (**C**) Extracellular gradients of cues are transduced to intracellular gradients of Ca^2+^ (middle panel) in the same direction as the extracellular gradients, and the increase in the intracellular Ca^2+^ level that depends on the ratio of cAMP to cGMP regulates the turning direction of the growth cone, *i.e*., attraction or repulsion. The biased direction of the effector distribution is thought to be reversed between attraction and repulsion (lower panel). High and low levels of intracellular Ca^2+^ induce attraction and repulsion, respectively, as indicated by the dotted arrows. (**D**) An activator-inhibitor system of intracellular signaling in chemotaxis: Guidance signal (G) regulates activator (A) and inhibitor (I) in turn up- and down-regulating the effector X. G, A, I and X correspond to Ca^2+^, CaMKII, PP1 and Rac1/Cdc42, respectively, in the growth cone. (**E**) Following exposure to a gradient of the guidance cue G, the gradients of A and I are formed across the growth cone, thereby the gradient of X is also formed. A^*^, I^*^ and X^*^ represent the levels of A, I and X, respectively, at the center of the growth cone. Δ*A*, Δ*I* and Δ*X* represent the spatial differences of A, I and X, respectively, across the growth cone. (**F**) The turning angle of the growth cone is formulated as Δ*X*/X^*^ based on the Weber-Fechner law. The balance between Δ*A*/A^*^and Δ*I*/I^*^ governs the turning direction, which is illustrated by scales: attraction when Δ*A*/A^*^> Δ*I* / I^*^ and repulsion when Δ*A*/A^*^ < Δ*I*/I^*^.

We developed a mathematical model of chemotactic cells based on intracellular signaling. The model chemotactic cell migrates and encounters extracellular gradient of a guidance cue (Fig. 1A). Though the cells have three-dimensional structures in reality, we addressed an intracellular one-dimensional (1D) coordinate, which is perpendicular to the migrating direction (Fig. 1A). Note that the cells are known to persistently migrate and turn according to polarized accumulation of intracellular signals along the coordinate perpendicular to the migrating direction^26^,^27^.

The model chemotactic cell is equipped with intracellular signaling molecules, the activator (A) and inhibitor (I), whose enzymatic activities are regulated by a guidance molecule, G (Fig. 1D). In this model, we did not specify the details of intracellular signaling, but assumed that the steady-state gradients of these intracellular signaling molecules, whose concentrations are denoted as *A* and *I*, are generated in accordance with the gradient of the extracellular guidance cue (Fig. 1E). This model is also equipped with a downstream effector X, which regulates the driving force for migration. X is directly up- and down-regulated, respectively, by the activator and inhibitor (Fig. 1D), and their regulations are assumed to be in reaction equilibrium state, in which its activity is locally determined by the ratio of *A* to *I*: *X(x*) = *αA(x*)/*I(x*), where *X* is the effector’s concentration, *x* denotes the one-dimensional coordinate of the model cell, and *α* is a positive constant. This assumption holds if the effector X is regulated by push-pull reaction (see Supplementary Information).

In the model, the migrating cell was turned based on spatial polarity of the distribution of X along the 1D coordinate, implying that X acted as a decoder that discriminated between attraction and repulsion. We here assumed that the downstream system that converts the spatial distribution of X into the growth cone turning response is endowed with adaptation property; this property was stated as the Weber-Fechner law, in which the detectable spatial polarity of X varies because of the scale of the concentration of X^28^. Indeed, the Weber-Fechner law has been found in several types of chemotactic cells^29^,^30^,^31^,^32^,^33^. We thus defined the turning angle, *ω*, of the growth cone as ΔX/X^*^, where X^*^denotes the effector’s level at the cellular center and Δ*X* is the spatial difference of X’s activity across the cell: Δ*X* = *X(L*/2)−*X*(−*L*/2), where *L* is the length of the cell (see Fig. 1A) and +*L*/2 and −*L/2* represent, respectively, the coordinates at the cell’s near and far sides with respect to the gradient of G. Thus, our model shows that if Δ*X* > 0, the cell is attracted and migrates toward the gradient; if Δ*X* < 0, the cell is repelled and turns away from the gradient.

Our model addressed only 1D coordinate, although the cells usually spread their morphology on a two-dimensional (2D) culture substrate. To further check the validity of our theory in a two-dimensional space, we also developed a 2D model (see Supplementary Figure 1 and Supplementary information) and confirmed that the migrating behaviors were consistent with those by the 1D model.

### Theory for chemotactic turning responses

In the presence of a shallow extracellular gradient of G, the turning angle *ω* is approximately derived as follows (see Methods)


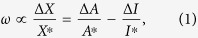


where A^*^ and I^*^ denote the levels of A and I, respectively, at the cellular center. Δ*A* and Δ*I* denote, respectively, the spatial differences of A and I across the cell: Δ*A* = *A(L*/2)−*A*(−*L*/2) and Δ*I* = *I(L*/2)−*I*(−*L*/2). Equation (1) indicates that the cellular migration direction depends on the balance between Δ*A*/A^*^ and Δ*I*/I^*^, *i.e.*, attractive (*ω* > 0) and repulsive (*ω* < 0) when Δ*A*/A^*^> Δ*I*/I^*^ and Δ*A*/A^*^ < Δ*I*/I^*^, respectively (Fig. 1F). This equation suggests that when the signs of Δ*A* and Δ*I* are opposite, the turning response is uni-directional (either attraction or repulsion) regardless of their magnitudes. For example, if Δ*A* is positive and Δ*I* is negative, only the attractive response occurs (Δ*X* > 0). This type of uni-directional chemoattractive response has commonly been observed in *Dictyostelium discoideum*, which is characterized by opposite intracellular gradients of PI3K and PTEN^5^. However, if the signs of Δ*A* and Δ*I* are the same, the migratory behaviors become bi-directional; switching occurs from attraction to repulsion and vice versa depending on the levels of A^*^ and I^*^. This type of phenomenon has typically been observed in nerve growth cones^13^,^15^,^18^,^34^,^35^,^36^. We subsequently focused on the bi-directional turning responses of growth cones.

### The model predicts both uni- and multi-phasic bi-directional turning responses

**Figure 2:**
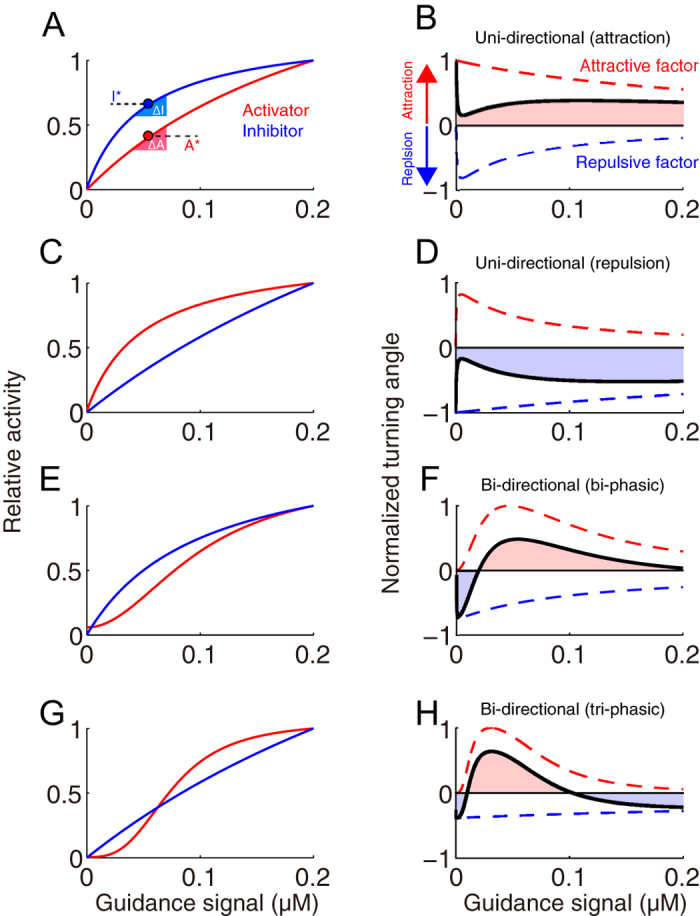
The theoretical model is capable of producing both uni-directional and multi-phasic bi-directional turning responses. (**A,C,E,G**) Examples of activator and inhibitor activities in response to increasing guidance signal (intracellular Ca^2+^ in the case of the growth cone). Red and blue lines indicate the dose-responses of activator A and inhibitor I, respectively, to the guidance signal. The dose-response curves are given by Hill equations (Equations (8 and 9)). Parameter values are listed in Methods. (**B,D,F,H**) Turning responses (black lines) are calculated based on the dose-responses of activator and inhibitor, shown in (**A,C,E,G**) (see Methods). When the intracellular Ca^2+^ level is in the red and blue shaded regions, the growth cone exhibits attraction and repulsion, respectively. The turning response is given by the sum of the attractive factor (red dashed line: the first term in Equation (1)) and the repulsive factor (blue dashed line: the second term in Equation (1)). Each dashed line represents the ratio between the slope and amplitude of the dose-response curve in (**B**), (**C**), (**E**) or (**G**); *e.g*., the red dashed line in (**B**) was obtained from the slope (Δ*A*) and amplitude (A^*^) of the red solid line in (**A**). Thus, the theoretical model is able to produce a simple uni-directional attraction (**A**,**B**) or repulsion (**C**,**D**), bi-phasic bi-directional turning response (**E**,**F**), and tri-phasic bi-directional turning response (**G**,**H**). These complex responses are primarily due to the nonlinear responses of the activator and inhibitor to the guidance signal, typically observed in (**G**).

In the growth cone, an extracellular gradient is converted to an intracellular Ca^2+^ gradient, whose direction is same as the extracellular gradient, and the level of Ca^2+^ regulates bi-directional responses (attraction or repulsion) (Fig. 1C)^15^. We here specifically examined how the intracellular Ca^2+^ level increase affects the turning response to the extracellular gradient. Our model assumes that an intracellular gradient of Ca^2+^ correlates with the gradient of the guidance signal G, and that the dose-responses of both activator A and inhibitor I are monotonically up-regulated by intracellular Ca^2+^ level (red and blue solid lines in Fig. 2A,C,E and G) and that Δ*A* and Δ*I* are proportional, respectively, to the dose-response slopes of A and I (see Equation (7) in Methods).

The model predicts different patterns of turning responses as the activities of A and I are progressively increased as the intracellular Ca^2+^ level increases: uni-directional responses, *i.e*., either attraction (Fig. 2B) or repulsion (Fig. 2D), and a bi-directional turning response, *i.e*., a change from repulsion to attraction (bi-phasic) (Fig. 2F). Interestingly, the model further predicts the existence of a tri-phasic bi-directional turning response, *i*.*e.*, a change from repulsion to attraction and then back to repulsion (Fig. 2H). As assumed in Fig. 2G, CaMKII and CaN/PP1, which function as an activator and inhibitor, respectively, in the growth cone, are well known to exhibit sigmoidal dose-responses to Ca^2+^ 37,38. Thus, this theoretical result suggests that the intracellular Ca^2+^ level would be responsible for growth cone’s bi-phasic and tri-phasic turning responses.

It should be noted that these model predictions were based on the turning angle Equation (1), which was derived based on the assumption of shallow extracellular gradient in the 1D model (Fig. 1A). Here, we checked if the 1D model reproduces the 2D model (Supplementary figure 1; see Supplementary Information) at least with a 10% extracellular gradient (i.e., 10% concentration difference between the near and far sides of the growth cone), typically used in the growth cone turning assay^39^,^40^. As a result, we found that our theory based on the 1D model well characterized the 2D migration of the growth cone under a biologically realistic range of gradient like 1–10%^41^,^42^,^43^,^44^,^45^ (Supplementary Figures 2 and 3).

### The cAMP/cGMP gradients induce tri-phasic bi-directional turning

**Figure 3:**
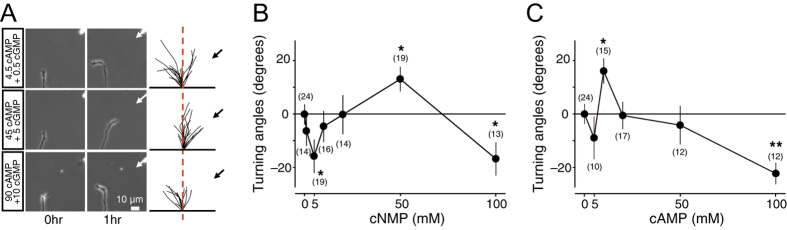
The growth cone exhibits multi-phasic bi-directional turning responses when the total concentration of [cAMP + cGMP] is changed at the fixed ratio of cAMP/cGMP. (**A**) Representative images of growth cones at the onset (0 hr) and after (1 hr) exposure to a gradient solution containing a mixture of the membrane permeable cyclic nucleotide analogues, [Sp-8-Br-cAMPS] and [8-Br-cGMP], at a fixed ratio of 9:1 at different concentrations (5 mM [4.5 cAMP + 0.5 cGMP]; 50 mM [45 cAMP + 5 cGMP]; and 100 mM [90 cAMP + 10 cGMP]) in the application micropipette. Superimposed traces on the right depict the total neurite trajectories examined over the 1 hr period, where the initial position of the growth cone is at the origin, and the original direction of growth is vertical. The arrows in both the representative images and trajectories mark the direction of the gradient. **(B)** The average growth cone turning angles in response to gradient of a mixture solution of [Sp-8-Br-cAMPS] and [8-Br-cGMP] with different concentrations (0, 1, 5, 10, 20, 50, and 100 mM) at the fixed ratio of 9:1 in the micropipette, which are indicated as “cNMP”. (**C**) The average growth cone turning angles in response to gradient of a solution of [Sp-8-Br-cAMPS] at different concentrations (5, 10, 20, 50, and 100 mM) in the micropipette. Positive and negative turning angles indicate attraction and repulsion, respectively. Error bars = s.e.m. (n) = number of growth cones examined. Significant differences compared to the control are indicated (*p < 0.05; **p < 0.01; Mann-Whitney U test).

To experimentally validate our theoretical prediction of growth cone bi-directional (both bi- and tri-phasic) turning responses (Fig. 2H), we performed *in vitro* turning assays of *Xenopus* spinal neuron growth cones^13^,^14^,^46^ by applying a gradient of the 9:1 ratio of the membrane permeable and phosphodiesterase (PDE)-resistant cyclic nucleotide analogues, Sp-8-Br-cAMPS and 8-Br-cGMP (Fig. 3); this ratio was previously observed to induce growth cone attraction^13^. Note that the use of PDE-resistant cyclic nucleotides allowed us to bypass ligand-receptor interactions^47^ and potential interdependent signaling events between these two cyclic nucleotides, such as the effects of PDEs^48^.

The growth cones were attracted by the gradient of a 9:1 ratio of cAMP:cGMP at a total concentration of 50 mM in application micropipettes (middle panel in Fig. 3A), as demonstrated previously^13^. However, the growth cones were repelled by the gradient at lower and higher total concentrations (5 mM and 100 mM). Thus, a tri-phasic bi-directional turning response was observed (Fig. 3B), implying that not only the ratio of cyclic nucleotide analogues but also the magnitude of their total concentration determines the growth cone turning direction. These results also suggested that a simple increase in attractive promoting factors (*e.g*., cAMP) or a decrease in repulsive promoting factors (*e.g*., cGMP) does not always result in either exclusive attraction or repulsion, respectively. In support of this observation, an external gradient of only Sp-8-Br-cAMPS, which is an attractive promoting factor, also induced tri-phasic bi-directional turning responses (Fig. 3C).

### Monotonic Ca^2+^ increase induces multi-phasic bi-directional turning

**Figure 4:**
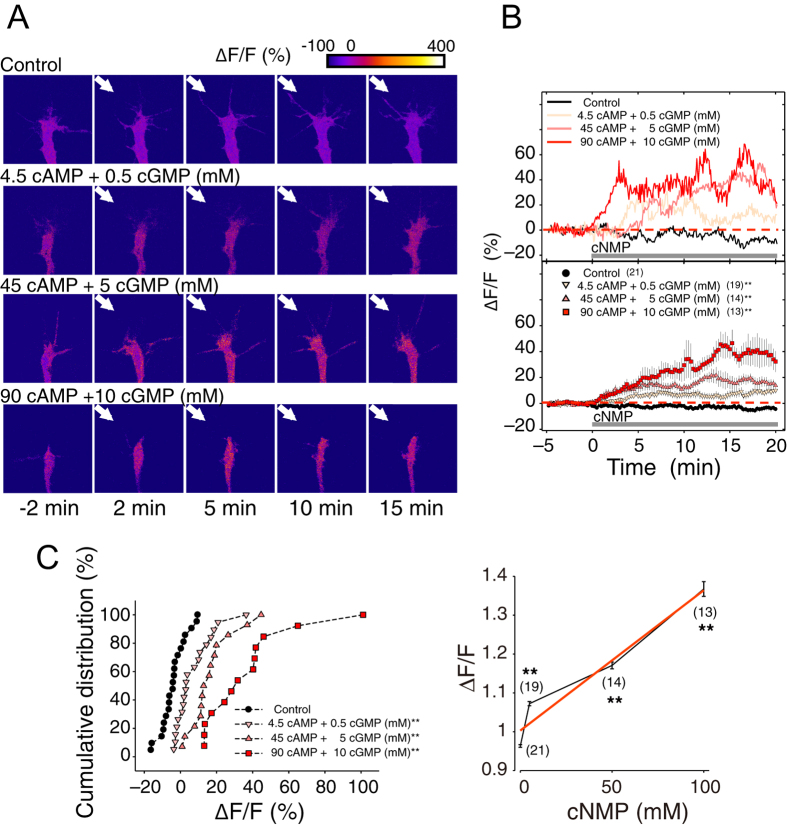
Multi-phasic bi-directional turning depending on the concentration of cAMP/cGMP gradients is accompanied by a monotonic Ca^2+^ increase. (**A**) Representative images of Oregon Green 488 BAPTA-1 (OGB)-dextran fluorescent growth cones obtained at various times during exposure to either culture medium (control) or gradients of different concentrations of a 9:1 ratio of cAMP/cGMP (arrows). The fluorescence intensity is expressed as pseudo-colors (top; blue = low, red = high). (**B**) Sample traces (top) and summary (bottom) of the percentage changes in OGB-dextran fluorescence (ΔF/F) in the growth cones exposed to culture medium (control) or to different concentration gradients of cAMP/cGMP. OGB-dextran fluorescence was normalized to the fluorescent intensity of co-injected Texas Red–dextran in the growth cones before and after exposure to the cyclic nucleotide gradients (arrows in (**A**)). Cyclic nucleotide (cNMP) gradients: grey horizontal bars. Error bars = s.e.m. (n) = number of growth cones examined. Significant differences compared to the control are indicated (*p < 0.05; **p < 0.01; Mann-Whitney U test). (**C**) Left panel: Cumulative distribution of the average ΔF/F (%) at 10–20 min after exposure to cyclic nucleotide gradients as in (**B**). Right panel: Monotonic increase in the averages ΔF/F at 15–20 min that resulted from growth cone exposure to cyclic nucleotide gradients as in (**B**). The red curve is a regression function.

We further examined the extent to which the growth cone Ca^2+^ increase is associated with the multi-phasic bi-directional turning behaviors by performing growth cone Ca^2+^ imaging in the presence of gradients of cyclic nucleotide analogues (Fig. 4A). Within several minutes of exposure to the gradients, the Ca^2+^ level in the growth cones increased and remained stable throughout the imaging procedure (Fig. 4B)^14^. Surprisingly, the Ca^2+^ response was monotonically increased as the amplitude of the gradients of cyclic nucleotide analogues increased (Fig. 4C). Furthermore, this Ca^2+^ imaging experiment demonstrated that repulsion is not only induced in response to a low growth cone Ca^2+^ increase, as previously reported^13^,^15^, but also occurs at a high Ca^2+^ increase. Taken together, the growth cone turning assays and the Ca^2+^ imaging results suggested that non-linear regulation of activator and inhibitor in the downstream signaling cascade induced by Ca^2+^ is responsible for the multi-phasic bi-directional turning behaviors of growth cones.

### System identification of CaMKII as an activator and PP1 as an inhibitor

**Figure 5:**
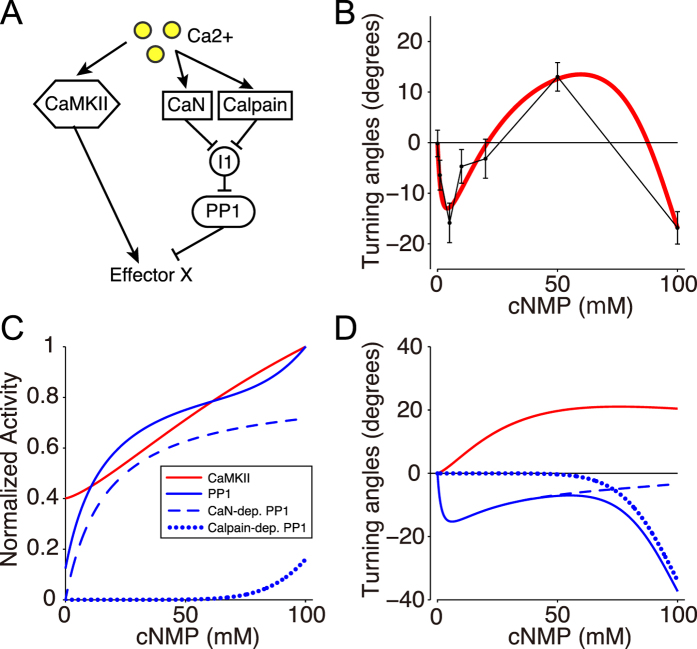
System identification of CaMKII and PP1 activities in the bi-directional turning response. (**A**) The schematic drawing depicting the regulation of CaMKII and PP1, which up- and down-regulate the effector X, respectively, in the growth cone model. (**B**) Simulation of the multi-phasic bi-directional turning response by a mathematical model where the parameters were identified by the reverse engineering approach (solid red line). Points and error bars represent the experimental results (the same as in Fig. 3B). Goodness of fitting was evaluated by the Pearson’s chi-square test (p = 0.78), indicating no significant difference. (**C**) Reverse-engineered dose-responses of CaMKII (Red line) and PP1 (blue line) to the total cAMP:cGMP concentration (cNMP). The dose-responses were normalized. The blue line is the sum of CaN- and Calpain-dependent PP1 (blue dashed and dotted lines). The estimated parameter values are listed in Methods. (**D**) Contributions of the activator (A; CaMKII) and the inhibitor (I; PP1) to growth cone turning (black line in (**B**)) depending on the cNMP level. Attractive and repulsive influences of the activator and inhibitor are represented by red and blue solid lines, respectively, which correspond to the first and second terms in Equation (1). The PP1-induced repulsion (blue solid line) is given by the sum of the CaN- and Calpain- dependent factors (dashed and dotted blue lines, respectively).

To identify how CaMKII (A; activator) and PP1 (I; inhibitor) are up-regulated in a Ca^2+^ -dose-dependent manner, we developed a mathematical model; the activity of CaMKII was expressed as a Hill equation of the Ca^2+^ concentration (Equation (10)), while the activity of PP1 was expressed as double Hill equations (Equation (11)) with lower and higher Kd values, which correspond to two distinct pathways: the CaN- and Calpain-dependent pathways, respectively (Fig. 5A), because higher level of Ca^2+^ is necessary for up-regulating Calpain than that for CaN^49^. We then took a reverse engineering approach (also called a system identification method), in which model parameters are estimated to fit the model into experimental data in general. Specifically, we optimized parameters of the Hill equations in terms of minimization of square error between experimental data of the tri-phasic turning responses (Fig. 3B) and model’s simulated turning responses as exemplified in Fig. 2, using a non-linear regression method (see Methods). The model with the estimated parameter values, called the reverse-engineered model, well reproduced the tri-phasic bi-directional turning responses of the growth cone (red line in Fig. 5B).

### Bi-directionality depends on the balance between CaMKII and PP1

**Figure 6:**
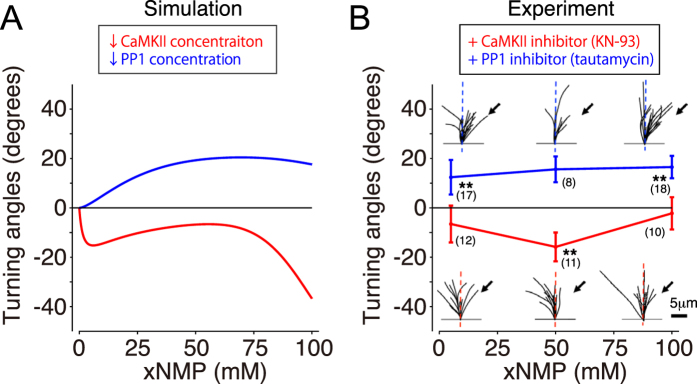
Bi-directional turning depends on the molecular switching between CaMKII and PP1. (**A**) Model simulation of growth cone turning responses during the inhibition of either the activator, CaMKII (red line) or the inhibitor, PP1 (blue line). Inhibition of CaMKII or PP1 activity was simulated by a 90% reduction of the estimated parameter for each concentration. (**B**) Growth cone turning responses were experimentally observed in response to a gradient solution that contained [Sp-8-Br-cAMPS] and [8-Br-cGMP] at a fixed ratio (9:1) at different concentrations in the presence of KN-93 (0.5 μM; CaMKII inhibitor, red line) or tautomycin (4 nM; PP1 inhibitor, blue line) in the assay medium. Significant differences compared to the control are indicated (*p < 0.05; **p < 0.01; Mann-Whitney U test). Goodness of fitting of model simulation to experiment was evaluated by p-value of the Pearson’s chi-square test (p < 0.01 with KN-93; p = 0.38 with tautomycin).

This reverse-engineered model prediction was further validated by the growth cone turning assays with the suppression of either CaMKII or PP1. First, we simulated the reverse-engineered model in which total CaMKII activity was reduced while other parameters were held constant, leading the prediction that the growth cones exclusively show repulsion with CaMKII suppression (red line in Fig. 6A). Next, we confirmed by growth cone turning assays with bath application of KN-93 (CaMKII inhibitor) that the tri-phasic bi-directional response observed in Fig. 3B became a repulsive uni-directional response (red line in Fig. 6B). Note that the reverse-engineered model was independent of data obtained from down-regulation experiments (Fig. 6B).

Alternatively, the simulated turning with the suppression of PP1 activity in the model resulted in the disappearance of the tri-phasic bi-directional turning response so that the model predicted only uni-directional attraction (blue line in Fig. 6A). Congruently, the growth cone turning assays with bath application of tautomycin (PP1 inhibitor) resulted exclusively in attraction (blue line in Fig. 6B). Therefore, the model predictions, together with the experimental results, supported the idea that the balance between the activator, CaMKII, and the inhibitor, PP1, determines whether the growth cone turning response is either uni-directional or bi-directional, and, furthermore, bi-phasic or tri-phasic bi-directional.

## Discussion

Intracellular Ca^2+^ signaling regulates growth cone bi-directional turning responses to many diffusible guidance cues. However, its molecular signaling mechanism is not well understood. In this study, we presented a mathematical model that identifies the growth cone chemotactic attraction and repulsion governed by the balance between the activator and inhibitor, as expressed by Equation (1) (Fig. 1F). We used both theoretical and experimental approaches to demonstrate how CaMKII and PP1, respectively, as activator and inhibitor, are non-linearly regulated depending on the intracellular Ca^2+^ and are responsible for multi-phasic growth cone turning in response to extracellular cues.

Our theoretical model was developed based on the intracellular signaling pathways shared by chemotactic cells. This model has the following characteristics: First, it assumes only “activator” and “inhibitor” as the intracellular signaling molecules that are commonly present in all chemotactic cells (Fig. 1D)^50^. Therefore, the theory is applicable not only to the growth cones, but also to the other chemotactic cells. Second, the theory is based on arbitrary gradients of the activator and inhibitor. Thus, it allows the detailed biochemical processes in intracellular signaling to be bypassed. Third, because this model only considers a few parameters, the reverse engineering approach was feasible (Fig. 5). These characteristics provide great advantage for identifying how the activator (*e.g*., CaMKII) and inhibitor (*e.g*., PP1) are regulated by an extracellular signal (Fig. 5) and the potential for experimental validation of the model (Fig. 6).

### Multi-phasic turning response

Our mathematical model generated various patterns of turning responses (uni- or bi-directional and bi- or tri-phasic) depending on the dose-response patterns of the activator and inhibitor (Fig. 2 and Supplementary figure 2). Such counterintuitive turning responses can be understood by our theory based on Equation (1); the turning response was determined by inequality between attractive factor (ΔA/A^*^) and repulsive factor (ΔI/I^*^) (see Δ*A,* ΔI, A^*^ and I^*^ in Fig. 2A). In Fig. 2E, for example, the attractive factor increased and then decreased when changing the guidance signal (red dashed lines), because its dose-response (A^*^) is a sigmoidally saturating function of the guidance signal, accompanied by transient changes of its slope (Δ*A*) as shallow, steep and then shallow. On the other hand, the repulsive factor monotonically decreased (blue dashed lines), because the dose-response (I^*^) and its slope (Δ*I*) are monotonically increasing and decreasing functions, respectively. Then, tug-of-war between these attractive and repulsive factors generated the bi-phasic bi-directional turning response (black line). In this way, our theory based on Equation (1) described the mechanism of turning responses, which was dependent on the dose-responses of A and I.

### Uni- and bi-directional gradient sensing

What is the critical difference between the uni-directional and bi-directional turning responses? Subsequently, we discuss their prerequisites.

Uni-directional gradient sensing: Our model can be expressed as a linear model equipped with an activator and inhibitor that exhibit linear dose-response patterns to a guidance signal G: *A* = *d*_*A*_*G* and *I* = *d*_*I*_*G*, where *d*_*A*_ and *d*_*I*_ denote positive sensitivity constants. The LEGI model exemplifies this type of linear model^6^. This linear model manifests a phenomenon called adaptation, in which the effector exhibits a constant steady-state response regardless of the magnitude of guidance gradients. This phenomenon occurs because of the constant nature of A^*^/I^*^. Δ*A* and Δ*I* are also constants that are determined by the diffusion coefficients of A and I, which are independent of the level of the guidance signal G^*^ (see Equation (S19) in Supplementary Information). In this linear model, the turning response *ω* in Equation (1) becomes *ω* = *c*_*A*_/*d*_*A*_G^*^−*c*_*I*_/*d*_*I*_G^*^, where *c*_*A*_ and *c*_*I*_ are positive constants defined in Methods. This equation states that the direction (sign) of the turning response is independent of the concentration of the guidance signal G. Thus, a chemotactic system with linear dose-response patterns would always exhibit uni-directional responses (either attraction or repulsion) in an adaptive manner. Then, what determines whether the uni-directional turning response is attractive or repulsive? Intuitively, when the inhibitor rapidly diffuses out, as assumed in the LEGI model, the inhibitor will be distributed almost uniformly over the growth cone with a small Δ*I*, which results in an attractive response (Δ*A*/A^*^ > Δ*I*/I^*^), and vice versa. A typical example of a chemotactic cell is *Dictyostelium discoideum*, which responds uni-directionally to a chemoattractant gradient at a wide range of concentrations (*e.g*., 5 pM-5 μM of extracellular cAMP)^51^.

Bi-directional gradient sensing: Our model typically expresses non-linear, sigmoidal-like responses of the activator and inhibitor as demonstrated in Figs 2 and 5. Because the dose-response of the effector X depends non-linearly on the concentration of guidance signal G, it no longer exhibits adaptation. The balance between Δ*A*/A^*^ and Δ*I*/I^*^ in Equation (1) thus can be switched from positive to negative and vice versa, resulting in bi-directional gradient sensing.

Therefore, our mathematical model can characterize the two basic and distinctive forms of chemotaxis: (1) a linear system with adaptation exhibits only uni-directional chemotaxis and (2) a non-linear system with non-adaptive character is required for exhibiting a bi-directional turning response, as observed in growth cones. Accordingly, there should be a trade-off between adaptation and bi-directionality.

### The reverse-engineered model

Our reverse-engineered model both qualitatively and quantitatively captured the tri-phasic bi-directional turning responses of the growth cone (no significant difference, p = 0.78, according to the Pearson’s chi-square test) (Fig. 5B). In the condition with pharmacological inhibition of CaMKII or PP1, the model’s predictive performance was also quantitatively evaluated, indicating that there was no significant difference with inhibition of PP1, but there was of CaMKII (p < 0.01 for CaMKII inhibition; p = 0.38 for PP1 inhibition according to the Pearson’s chi-square test). However, the model still qualitatively predicted actual growth cone turning even in these conditions; the model actually reproduced uni-directional repulsion and attraction with inhibition of CaMKII and PP1, respectively (Fig. 6).

In the reverse-engineered model, PP1 was up-regulated by two distinct CaN- and Calpain-dependent pathways. In the CaN-dependent pathway, Ca^2+^ -bound Calmodulin (CaM) up-regulates CaN, which subsequently up-regulates PP1 by the suppression of Inhibitor 1 (I1)^52^. In the Calpain-dependent pathway, higher level of Ca^2+^ than that required for CaN activation up-regulates Calpain^49^, which converts p35 to p25 and causes prolonged activation of cyclin dependent kinase 5 (Cdk5), which then inhibits I1 and subsequently up-regulates PP1^53^,^54^. In addition, CaMKII and PP1 were independently addressed in the model though PP1 was known to inhibit CaMKII^55^. On the other hand, Ca^2+^ -dependent dose-response of CaMKII was a monotonically increasing function even with PP1, while PP1 affected its Kd value^56^. Thus, CaMKII dose-response to Ca^2+^ in the model included the effect of PP1.

The reverse-engineered model elucidated the following underlying mechanisms responsible for inducing multi-phasic bi-directional turning responses (Fig. 5C–D): (1) A low level of Ca^2+^ increase favorably triggers the CaN-dependent activation of PP1 over either the Calpain-dependent activation of PP1 or CaMKII, which then induces repulsion. (2) A moderate Ca^2+^ increase favorably triggers CaMKII over CaN- and Calpain-dependent activation of PPI, which then induces attraction. Importantly, we argue that the Ca^2+^ increase associated with growth cone attraction is considerably lower (*i.e*., 100 to 200 nM)^17^,^57^,^58^ than the extent to which Ca^2+^ recruits the autophosphorylation of CaMKII^24^,^59^,^60^, suggesting that CaMKII autophosphorylation is not a critical requirement for growth cone bi-directional turning behaviors. (3) A high Ca^2+^ increase strongly induces the Calpain-dependent activation of PP1, while sustaining the activation of both CaMKII- and CaN-dependent PP1. Consistently, a previous study indicated that Calpain activation requires a high level of Ca^2+^ (>10 μM)^49^, although this high level of Ca^2+^ increase was not normally induced by guidance cues^14^,^15^,^57^,^58^,^61^. Increasing evidence has indicated that Calpain activation can occur at physiological Ca^2+^ concentrations^62^,^63^,^64^. Thus, the modulation of growth cone repulsion by the Calpain-PP1 pathway may occur under some physiological conditions.

### Comparison with previous computation models

There have been many studies of computational models for chemotaxis. Most of them were interested in gradient sensing mechanisms with an emphasis on non-neural cells, *e.g*., *Dictyostelium discoideum*^6^,^7^,^8^,^9^,^65^,^66^,^67^,^68^,^69^,^70^. These models intended primarily to describe uni-directional attractive gradient sensing by means of global inhibition, *e.g*., a rapidly diffusing inhibitor. For example, the LEGI model^6^,^7^,^9^,^70^ elucidates the mechanism of “adaptation”, in which chemotactic cells exhibit greater sensitivity to the gradient of a chemoattractant while exhibiting insensitivity to the magnitude of the chemoattractant gradient. On the other hand, our model showed that non-adaptive character is required for realizing bi-directional turning responses.

The growth cone has also been computational modeled by several aspects^71^. Motivations to model the growth cone cover formation of the extracellular gradient that the growth cone senses^42^,^72^,^73^, axonal pathfinding by the gradient cone^74^,^75^,^76^,^77^,^78^,^79^,^80^, the growth cone movement in three-dimensional space^25^,^81^,^82^,^83^, axonal specification during neuronal polarization^84^,^85^,^86^,^87^,^88^ and the gradient sensing based on intracellular reaction^23^,^25^,^74^,^89^ or on Bayesian information approach^90^,^91^.

Related to our current study, Forbes *et al.*^23^ and Roccasalvo *et al.*^25^ addressed bi-directional turning responses of the growth cone based on models of intracellular Ca^2+^ signaling. While these models were specialized in the growth cone, we also intended to establish the general theory that describes the mechanistic difference between uni-directional chemotaxis of non-neural cells and bi-directional chemotaxis of growth cones. In Roccasalvo *et al.*, intracellular Ca^2+^ dynamics was described as a 2D reaction-diffusion system which was based on the Gierer-Meinhartd model^74^,^89^ with Turing instability^92^. The growth cone was turned according to spatial polarity of Ca^2+^ in their model. The turning response was, on the other hand, determined by spatial polarity of Ca^2+^ downstream effector, *e.g*., Rac1 and Cdc42, in our 1D model, in which the spatial polarity was represented by sign and magnitude of Δ*X*; the latter is natural, because the study of growth cone turning assay using Ca^2+^ uncaging showed that attraction and repulsion were both induced by intracellular Ca^2+^ elevation with the same polarity, but attraction or repulsion was determined by the elevation level^18^. In addition, other Ca^2+^ imaging studies clarified that intracellular Ca^2+^ constitutes the gradient of the same direction as the extracellular gradient during both attraction and repulsion^15^. Moreover, Rac1 and Cdc42 participate in membrane protrusion of the growth cone^93^,^94^ via regulating actin dynamics of filopodia and lamellipodia^95^,^96^, regulated by Ca^2+^-dependent CaMKII and PP1^22^,^97^,^98^. Thus, as addressed in our model, Rac1 and Cdc42 in the Ca^2+^ downstream should be important determinants for the growth cone turning. In comparison to the existing 2D model, our 1D model is advantageous, because it has the ability to explain the basic mechanism in the molecular level that allows the growth cone to exhibit bi-directional and even multi-phasic turning behaviors with the help of simple linear approximation. We also confirmed simple extension of our theory to 2D, i.e., a 2D model, well showed turning behaviors of the growth cone (Supplementary Figure 2).

Forbes *et al.* simulated Ca^2+^ signaling pathway including CaMKII, CaN, and PP1 in the growth cone and described the tri-phasic bi-directional turning responses; their model was an extension of existing model for signaling pathway in synaptic plasticity^24^, reflecting the fact that CaMKII, CaN, and PP1 are also equipped in a dendritic spine^99^,^100^. In contrast to our model with PP1 as an inhibitor, Forbes *et al.*’s model considered CaN as an inhibitor and PP1 as an inhibitory modulator of CaMKII. Their model predicted that activity changes of PKA, which negatively regulates PP1, induce shift of the turning response depending on Ca^2+^, still maintaining the bi-directionality. On the other hand, our growth cone turning assays with suppression of PP1 showed that the bi-directionality disappeared and only attraction was induced (Fig. 6B). This experimental result has clearly shown that the molecular tug-of-war between PP1 and CaMKII is crucial for tri-phasic bi-directional turning. In addition, CaMKII possesses bi-stability in their model, in which the model’s intracellular signaling was adopted from a previous model with CaMKII bi-stability^24^. It should be noted here that CaMKII has sometimes been hypothesized to function as a bi-stable memory element at postsynaptic spines during the induction of long-term potentiation^24^,^59^,^60^,^101^, while such bi-stable character has not been observed by an *in vivo* imaging of CaMKII activity in dendritic spines^102^. Related to this controversy, our reverse-engineered model suggested monotonical dose-response of CaMKII, instead of more complicated one like with hysteresis; the former never produces bi-stability.

## Methods

### Theoretical methods

#### Chemotactic turning response

The chemotactic cells have been known to detect shallow extracellular gradients (*e.g*., few percent difference of concentrations in *Dictyostelium discoideum*^51^,^103^ and the growth cone^41^,^42^,^43^,^44^,^45^). Then, the chemotactic turning response is modeled under a shallow extracellular gradient. In response to the extracellular gradient, intracellular gradients of A and I, *A(x*) and *I(x*), are supposed to be produced in a shallow manner across the growth cone (Fig. 1E). In this situation, it can be considered that at a specific position *x*′ on the cellular coordinate, the activities of A and I, *A(x* = *x*′) and *I(x* = *x*′), are slightly perturbed from their activities at the cellular center *x* = 0, i.e., *A(x* = 0) and *I(x* = 0), respectively. Due to the assumption of shallowness, the activity of X at the position *x*′, *X*′, can be approximately linearized as


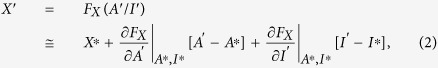


where *F*_*X*_(*A/I*) represents the activity of X given a ratio of *A* to *I*; *A*′, *I*′ and *X*′ indicate *A(x* = *x*′), *I(x* = *x*′), *X(x* = *x*′), respectively; A^*^, I^*^ and X^*^ indicate *A(x* = 0), *I(x* = 0) and *X(x* = 0), respectively. Here, the first-order Taylor expansion with respect to *A*′ and *I*′ around A^*^ and I^*^ was used, where *A*′−A^*^ and *I*′−I^*^ represent small perturbations. Because the position *x*′ can be arbitrarily selected, this equation can be generalized for any position *x* as


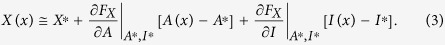


The spatial difference of *X* across the cell was simply calculated by Δ*X* = *X(L*/2)−*X*(−*L*/2), where *L* indicates the length of the cell; *L*/2 and −*L*/2 indicate the spatial coordinates at two ends facing higher and lower extracellular guidance signal, respectively (Fig. 1A); *X(L*/2) and *X*(−*L*/2) were obtained by substituting *L*/2 and −*L*/2 for *x* in Equation (3), respectively. Then, Δ*X* becomes


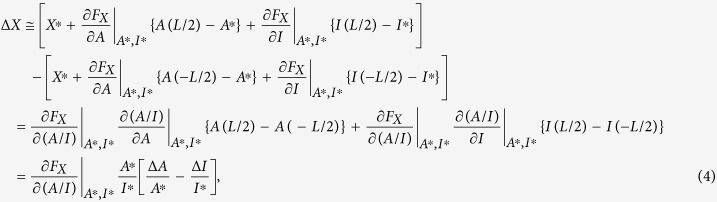


where Δ*A* and Δ*I* indicate the spatial differences of the activator and inhibitor, respectively, across the growth cone coordinate (Fig. 1E). We also derived Equation (4) in different way (see Supporting Information).

In the model, the turning angle *ω* of the chemotactic cell was proportionally determined by ΔX/X^*^ according to the Weber-Fechner law, which states that alterations of a detectable gradient are based on the scale of the concentration X^*^:


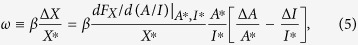


where *β* denotes a positive constant (the coefficient of Equation (1)). Note that the sign of the turning angle *ω* (attraction or repulsion) is not affected even if *X* non-linearly depends on *A*/*I*, though the amplitude is changed due to the non-linearity. If *X* is proportional to *A*/*I* (*F*_*X*_(*A*/*I*) = *αA*/*I*),


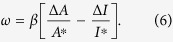


Note that in Equation (6), *α* was cancelled out.

#### The turning response to the extracellular gradient

Based on Equation (6), we evaluated the turning angle in response to an extracellular gradient *G(x*). Suppose that the activities of A and I are regulated by the guidance signal G in a dose-dependent manner as *A* = *F*_*A*_(*G*) and *I* = *F*_*I*_(*G*), respectively (Fig. 2A,C,E,G). A^*^ and I^*^ in Equation (6) can be replaced by *F*_*A*_(G^*^) and *F*_*I*_(G^*^). Δ*A* and Δ*I* in Equation (6) were simply set to be proportional to the derivatives of *F*_*A*_(*G*) and *F*_*I*_(*G*) with respective to *G*, i.e., *dF*_*A*_/*dG*_G*_ and *dF*_*I*_/*dG*_G*_, respectively. Then, Equation (6) was rewritten by


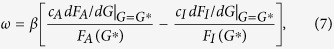


where *c*_*A*_ and *c*_*I*_ are positive constants which describe the sensitivity of A and I to the extracellular gradient, depending on their diffusion constants. Note that the assumption used here (Δ*A* = *c*_*A*_*dF*_*A*_/*dG*_G*_ and Δ*I* = *c*_*I*_*dF*_*I*_/*dG*_G*_) can result from intracellular reaction-diffusion dynamics of A and I (see Supporting Information).

#### Activator-inhibitor dose-response model

In Fig. 2, the dose-response curves of the activator (A) and inhibitor (I) were given by Hill equations:


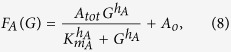



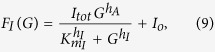


where *A*_*tot*_ and *I*_*tot*_ indicate maximum activities of A and I, respectively; *A*_*o*_ and *I*_*o*_ indicate basal activities of A and I, respectively; *h*_*a*_ and *h*_*I*_ are Hill coefficients of the dose-responses of A and I, respectively, which regulate the non-linearity of sigmoidal curves; *Km*_*A*_ and *Km*_*I*_ indicate Ca^2+^ concentrations required for half activation of A and I, respectively. Parameter values used in the Hill equations above, to draw the Fig. 2’s panels, are listed below:

Figure 2A: *A*_*tot*_ = 1, *h*_*A*_ = 1, *Km*_*A*_ = 0.25, *A*_*o*_ = 0, *I*_*tot*_ = 1, *h*_*I*_ = 1, *Km*_*I*_ = 0.05, *I*_*o*_ = 0.01.

Figure 2C: *A*_*tot*_ = 1, *h*_*A*_ = 1, *Km*_*A*_ = 0.05, *A*_*o*_ = 0.01, *I*_*tot*_ = 1, *h*_*I*_ = 1, *Km*_*I*_ = 0.5, *I*_*o*_ = 0.

Figure 2E: *A*_*tot*_ = 1, *h*_*A*_ = 2, *Km*_*A*_ = 0.1, *A*_*o*_ = 0.05, *I*_*tot*_ = 1, *h*_*I*_ = 1, *Km*_*I*_ = 0.1, *I*_*o*_ = 0.001.

Figure 2G: *A*_*tot*_ = 1, *h*_*A*_ = 3, *Km*_*A*_ = 0.075, *A*_*o*_ = 0.1, *I*_*tot*_ = 1, *h*_*I*_ = 1, *Km*_*I*_ = 0.5, *I*_*o*_ = 0.01.

In Fig. 5A, the activities of CaMKII (A) and PP1 (I) are expressed by dose-response functions of the Ca^2+^ concentration *C*:


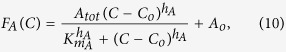



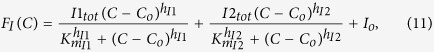


where *A*_*tot*_, *I1*_*tot*_ and *I2*_*tot*_ indicate maximum activities of CaMKII, CaN-dependent PP1 and Calpain-dependent PP1, respectively; *A*_*o*_ and *I*_*o*_ indicate basal activities of CaMKII and PP1, respectively; *h*_*a*_, *h*_*I1*_ and *h*_*I2*_ are Hill coefficients of the dose-responses of CaMKII, CaN-dependent PP1 and Calpain-dependent PP1, respectively; *Km*_*A*_, *Km*_*I1*_ and *Km*_*I2*_ indicate Ca^2+^ concentrations required for half activation of CaMKII, CaN-dependent PP1 and Calpain-dependent PP1, respectively. The dose-response of CaMKII is expressed as a Hill equation with the Ca^2+^-independent basal activity *A*_*o*_, whereas that of PP1 consists of double Hill equations with the basal activity *I*_*o*_. The first and second right-hand-side terms of Equation (11) correspond to activations by CaN- and Calpain-dependent PP1 pathways, respectively. *C*_*o*_ is the basal Ca^2+^ concentration.

The Ca^2+^ concentration *C* is also a function of the total concentration of the cyclic nucleotide analogue, *N*, which is represented as *C(N*). In this model, *C(N*) was obtained by a fitting function based on the Ca^2+^ imaging data as 0.0036110*N* + 1.00308 (right panel in Fig. 4C). The turning angle *ω* was calculated as described above with the following minor modification. Δ*A* and Δ*I* in Equation (6) were assumed to be proportional, respectively, to both the derivatives of *A* and *I* with respect to *N*, and to the total concentration of cyclic nucleotide analogue *N*, as Δ*A* = *c*_*A*_*N(dF*_*A*_/*dC*)(*dC*/*dN*) and Δ*I* = *c*_*I*_*N(dF*_*I*_/*dC*)(*dC*/*dN*). Because the slope of the extracellular gradient of cyclic nucleotide analogue in the experimental paradigm should depend proportionally on its applied concentration and this extracellular gradient is translated into the intracellular Ca^2+^ gradient, Δ*A* and Δ*I* should be proportional to the total concentration of the cyclic nucleotide analogue.

Parameters of Equations (7, 10 and 11) were estimated by a reverse-engineered approach and used for depicting Fig. 5. Those values were:

*γ* = 42.20511, *c*_*A*_ = 1, *c*_*I*_ = 0.76617, *A*_*tot*_ = 1, *h*_*A*_ = 1.35144, *Km*_*A*_ = 0.48443, *A*_*o*_ = 0.26922, *I1*_*tot*_ = 1, *h*_*I1*_ = 1.00000, *Km*_*I1*_ = 0.061014, *I2*_*tot*_ = 1.40063, *h*_*I2*_ = 7.59427, *Km*_*I2*_ = 0.46046, *I*_*o*_ = 0.14789, *C*_*o*_ = 1.00308

#### Estimation of parameters

The parameters, *Km*_*A*_, *h*_*A*_, *A*_*o*_, *Km*_*I1*_, *h*_*I1*_, *I2*_*tot*_, *Km*_*I2*_, *h*_*I2*_, *I*_*o*_ in Equations (10 and 11) and *γ*, *c*_*I*_ in Equation (7), which are denoted in total as Θ, were estimated by a non-linear regression to minimize the quadratic error function as follows:


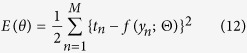


where *f(y*_*n*_; Θ) is a function that analytically describes the turning angle of the growth cone based on Equation (7), *y*_*n*_ and *t*_*n*_ denote the input variable (total concentration of Sp-8-Br-cAMPS and 8-Br-cGMP) and the target variable (turning angle of the growth cone), respectively, of the *n*th data sample obtained by the turning assays, and *M* indicates the total number of data samples. Note that *A*_*tot*_, *I1*_*tot*_ and *c*_*A*_ were not optimized and set to 1 and *C*_*o*_ was set to basal value of Δ*F*/*F* (right panel in Fig. 4C). We used a simplex Nelder–Mead algorithm to minimize the error function (12). Although this optimization had an issue of local minima because of its non-linearity regarding the parameters to be estimated, we repeated its solution many times by changing its initial condition and then selected the best fit.

### Experimental methods

#### Neuronal cultures

Cultures of *Xenopus* spinal neurons were prepared from neural tubes of stage 22 embryos and were used for growth cone turning assays and Ca^2+^ imaging at 14–18 h after incubation at 23–25 °C as previously described^13^,^14^,^46^. The culture medium consisted of 49% (v/v) LB medium (Gibco), 1% (v/v) FBS (HyClone) and 50% (v/v) Ringer’s solution (in mM: 115 NaCl, 2 CaCl_2_, 2.5 KCl and 10 HEPES (pH 7.4)). All experiments were in accordance with protocols approved by the Institutional Animal Care and Use Committee of the New York University School of Medicine.

#### Chemical gradients and pharmacological usage

The membrane-permeable analogues of cNMP, CaMKII inhibitor (KN-93) and PP1 inhibitor (tautomycin) were purchased from Calbiochem. The microscopic gradients of the membrane-permeable analogues of the cNMP solutions were generated through a micropipette with a tip opening of 1 μm by applying repetitive pressure ejection, as described previously^14^. Pharmacological inhibitors (KN-93: 0.5 μM; tautomycin: 4 nM) were applied in the culture medium (4 ml total volume) at least 30 min before the start of each experiment and were present throughout the experiments.

#### Growth cone turning assay

The growth cone turning assay was accomplished as described previously^13^,^14^,^46^. Briefly, a micropipette tip that contained the chemical solutions was placed 100 μm away from the palm of the growth cone at an angle of 45° with respect to the initial direction of neurite extension (indicated by the last 10-μm segment of the neurite). Images of both the initial and final growth cones were recorded using a charge-coupled device (CCD) camera (Hitachi KP-M2U) attached to a phase contrast microscope (Olympus CKX-41). Growth cones with a net extension >10 μm over the 1-h period were analyzed using NIH ImageJ software. The final turning angle (expressed in degrees), which represents the angle between the initial direction of the neurite extension and a straight line connecting the positions of the growth cone at the onset and the end of the 1-h exposure to the cNMP solutions, was measured.

#### Calcium imaging

Calcium imaging of the growth cones was performed as described previously^14^. The soma of isolated *Xenopus* spinal neurons were microinjected with 200 μM Oregon Green 488 BAPTA-1 and Texas Red conjugated to 10-kDa dextran (Molecular Probes, Inc.) at least 3 hours before the imaging, using an Eppendorf pressure injection system (Transjector 5246)^15^,^16^. Calcium imaging was performed using a Yokogawa confocal system (CSU-22, Perkin Elmer) equipped with an Ar/Kr gas laser. Excitation at 488 nm and 568 nm was controlled by an acousto-optical tunable filter (AOTF), and Oregon green BAPTA and Texas red fluorescence emission signals were collected, respectively, at 520–540 nm and 614–642 nm by an EM-CCD camera (Hamamatsu) through a 100× objective (UPlanSApo, N.A. 1.4, Olympus). Fluorescence images were collected sequentially in pairs every 5 s and analyzed using UltraView (Perkin Elmer) and ImageJ software. The mean fluorescence intensity at a growth cone was measured over an area that covered the entire growth cone. Oregon Green fluorescence was normalized to Texas Red fluorescence to control for experimental fluctuations, *e.g*., growth cone volumes or focal plane changes. The fluorescence ratio at each sampling time was normalized to the average fluorescence ratio measured during the initial 5-min baseline period.

## Additional Information

**How to cite this article**: Naoki, H. *et al.* Multi-phasic bi-directional chemotactic responses of the growth cone. *Sci. Rep.* **6**, 36256; doi: 10.1038/srep36256 (2016).

**Publisher’s note:** Springer Nature remains neutral with regard to jurisdictional claims in published maps and institutional affiliations.

## Supplementary Material

Supplementary Information
