# Supplementary material for: Multi-phasic bi-directional chemotactic responses of the growth cone
Source: Sci Rep. 2016 Nov 3;6:36256. doi: 10.1038/srep36256 (PMC5093620; doi:10.1038/srep36256)
Supplement: Supplementary Information [file srep36256-s1.pdf]

Supplementary Information

Multi-phasic bi-directional chemotactic responses of the growth cone

Honda Naoki[1,2], Makoto Nishiyama[3,4], Kazunobu Togashi[3], Yasunobu Igarashi[5], Kyonsoo Hong[3,4] and Shin Ishii[2,6]

1. Graduate School of Medicine, Kyoto University, Sakyo, Kyoto, Japan
2. Imaging Platform for Spatio-temporal Information, Kyoto University, Sakyo, Kyoto, Japan
3. Department of Biochemistry, New York University School of Medicine, New York, New York, USA
4. Kasah Technology Inc. New York, New York, USA
5. Olympus Software Technology Corporation, Hachioji, Tokyo, Japan
6. Graduate School of Informatics, Kyoto University, Sakyo, Kyoto, Japan

Corresponding author for theory: Honda Naoki
Address: Building F 301, Yoshidakonoe, Sakyo, Kyoto, Japan 606-8315
Tel: +81-75-753-4422
Fax: +81-75-753-4698
E-mail: n-honda@sys.i.kyoto-u.ac.jp

Corresponding author for experiment: Kyonsoo Hong
Address: Kasah Technology Inc., 340 East 23rd Street, New York, NY 10010, USA
Tel: 646-416-4530
Fax: 646-692-9465
E-mail: Kyonsoo.hong@kasahtechnology.com

Competing Financial Interests: NO

Effector regulation by push-pull reaction

To present physical implementation by which the effector X's activity is determined by the ratio of activator A and inhibitor I, we considered enzymatic push-pull reaction, which ubiquitously exists in intracellular signaling. The dynamics of the concentration of active X, X, are simply described by

$$\frac{dX}{dt} = k_A(X_{tot} - X)A - k_I XI, \tag{S1}$$

where X_{tot} is the total concentration of the effector X, and k_A and k_I represent the catalytic reaction rates of activator and inhibitor, respectively. The steady state of X is determined by the ratio A/I as

$$X_{eq} = \frac{X_{tot}(A/I)}{(k_I/k_A) + (A/I)}. \tag{S2}$$

When $k_I/k_A >> A/I$, the steady state of X can be approximately proportional to A/I as $X=\beta A/I$, where $\beta=X_{tot}(k_A/k_I)$. If the enzymatic activities of A and I are modeled by Michaelis-Menten equation, the dose-response of X also increases as the ratio A/I increases typically in a sigmoidal manner[1,2].

Another derivation of equation (4)

Equation (4) in the main text can be derived by different way. Given $A(x)$ and $I(x)$, distribution of X is determined by $X(x)=F_X(A(x)/I(x))$. Here, we evaluated slope of the distribution of X at x=0 as

$$\begin{aligned} \left.\frac{dX}{dx}\right|_{x=0} &= \left.\frac{dF_X}{d(A/I)}\right|_{x=0} \left.\frac{d(A/I)}{dx}\right|_{x=0} \\ &= \left.\frac{dF_X}{d(A/I)}\right|_{x=0} \left(\left.\frac{\partial(A/I)}{\partial A}\right|_{x=0} \left.\frac{dA}{dx}\right|_{x=0} + \left.\frac{\partial(A/I)}{\partial I}\right|_{x=0} \left.\frac{dI}{dx}\right|_{x=0} \right) \\ &= \left.\frac{dF_X}{d(A/I)}\right|_{x=0} \frac{A(x=0)}{I(x=0)} \left(\frac{1}{A(x=0)} \left.\frac{dA}{dx}\right|_{x=0} - \frac{1}{I(x=0)} \left.\frac{dI}{dx}\right|_{x=0} \right) \end{aligned} . \tag{S3}$$

If $A(x)$, $I(x)$ and $X(x)$ are assumed to be linear function of x, their spatial differences across the cell can be represented $\Delta A=dA/dx_{x=0}L$, $\Delta I=dI/dx_{x=0}L$ and $\Delta X=dX/dx_{x=0}L$, where L denotes the length of the cell. Multiplication of equation (S3) by L leads to

$$\begin{aligned} \Delta X &= \left.\frac{dF_X}{d(A/I)}\right|_{x=0} \frac{A(x=0)}{I(x=0)} \left(\frac{\Delta A}{A(x=0)} - \frac{\Delta I}{I(x=0)} \right) \\ &= \left.\frac{dF_X}{d(A/I)}\right|_{A^*,I^*} \frac{A^*}{I^*} \left[\frac{\Delta A}{A^*} - \frac{\Delta I}{I^*} \right] \end{aligned}, \tag{S4}$$

where A^* and I^* indicate $A(x=0)$ and $I(x=0)$, respectively. This equation is the same as equation (4) in the main text.

In this derivation, linearity of $A(x)$, $I(x)$ and $X(x)$ were imposed, which are stronger assumptions than that in the main text. Actually, equation (4) was derived with the assumption that $A(x)$ and $I(x)$ were given as being slightly perturbed from $A(x=0)$ and $I(x=0)$, respectively, no matter if $A(x)$ and $I(x)$ were linear or non-linear function of x. Therefore, the derivation of equation (4) is more general than that of equation (S4).

2D model

To confirm the validity of our theoretical consideration based on the 1D model, we extended our 1D model to a 2D model, which enabled simulation of 2D migrations of the growth cone. The model growth cone was represented by an ellipse-shaped body as in Roccasalvo *et al.*[3] and received an extracellular gradient of the guidance signal.

Extracellular gradient

We used a realistic gradient typically used in the growth cone turning assay, in which chemical solution was repeatedly ejected with 2 Hz ejected from a micropipette located at 100 μm distant from the center and 45° from the migrating direction of the growth cone[4,5] (**Supplementary figure 1A and B**). The gradient in the 2D space was described by summation of Gaussian kernels[4] as

$$G(\mathbf{r}) = \sum_{j=1}^{t/t_o} \frac{A}{4\left[\pi D(t - jt_o)\right]^{3/2}} \exp\left[-|\mathbf{r} - \mathbf{r}_o|^2 / 4D(t - jt_o)\right], \tag{S5}$$

where A, $\mathbf{r}$, $\mathbf{r_o}$ D, j, t and t_o indicate concentration of the chemical solution at the source, positional vector in the 2D space as $\mathbf{r}=(r_x, r_y)^T$, 2D position of the chemical source (location of the micropipette), diffusion constant of the chemical, order (index) of the repetitive ejections, elapsed time from the initial application, time interval of the repetitive ejections, respectively. Parameter values used in equation (S5) were t=3600 (s), t_o=1/2 (s), D=520 (μm^2/s) [4] and $\mathbf{r_o}=(100/(2^{1/2}), 100/(2^{1/2}))^T$ (μm). Because the chemical gradient is known to become almost stationary after 10 min [4], we used t=3600 (s).

Intracellular signals in response to extracellular gradient

The guidance signal up-regulated activator and inhibitor according to their dose-response functions, $A(G(\mathbf{r}))$ and $I(G(\mathbf{r}))$, respectively (**Supplementary figure 1C**). The activity of the effector X was also locally determined by a ratio of the activator to inhibitor as $X(\mathbf{r})=A(G(\mathbf{r}))/I(G(\mathbf{r}))$ (**Supplementary figure 1D**).

Migration of the growth cone regulated by X

In the 2D model, biased distribution of X generates the turning force such that X along periphery of the growth cone locally generates protrusive force in normal direction (perpendicular to tangential line of the growth cone), and the summation of these protrusive forces drives the growth cone turning behavior (**Supplementary figure 1D**). We assumed that the downstream system that converts the spatial distribution of X into the growth cone turning force is endowed with adaptation property; this property is known as the Weber-Fechner law[6], in which the detectable biased distribution of X varies because of the scale of the spatial average of X. Thus, the turning force vector was described by

$$\mathbf{F}_{turn}(t) = \gamma \frac{\oint_C \mathbf{n}(t,s) X(t,s)\, ds}{\oint_C X(t,s)\, ds \Big/ S}, \tag{S6}$$

where γ, s, S, $X(s)$, and $\mathbf{n}(t, s)$ indicate a positive constant, the 1D coordinate along the periphery of the growth cone, perimeter of the growth cone, the activity of X at coordinate s along the periphery and a unit normal vector at coordinate s along the periphery at time t, respectively. The integrals in equation (S6) are

line integrals, in which the functions, *e.g.*, $\mathbf{n}(t, s)X(t, s)$ and $X(t, s)$ to be integrated, were evaluated along the periphery of the growth cone. The numerator represents the sum of the local protrusive forces driven by X, whereas the denominator represents the average level of X.

In addition to the turning force, the model growth cone was driven by the persistent force, which was constitutively generated, being independent of X (**Supplementary figure 1D**). In fact, the growth cone persistently migrates even without the extracellular gradient. The motion of the growth cone was then described by

$$\frac{d\mathbf{r_c}}{dt} = \mathbf{F}_{mig}, \tag{S7}$$

where $\mathbf{r_c}$ indicates the 2D positional vector of the center of the growth cone ($\mathbf{r_c}=(r_{cx}, r_{cy})^{\mathrm{T}}$) and $\mathbf{F}_{mig}$ represents total migration force vector consisting of the two kinds of forces as

$$\mathbf{F}_{mig}(t) = v\mathbf{p}(t) + \mathbf{F}_{turn}(t), \tag{S8}$$

where $v\mathbf{p}$ represents the persistent force vector whose amplitude (norm) and direction (unit vector) are denoted by v and $\mathbf{p}$, respectively. Note v is a parameter representing the migration speed of the growth cone even without the extracellular gradient. The direction of the persistent force vector, $\mathbf{p}$, was also modulated by direction of the migration, i.e., $\mathbf{F}_{mig}$, as

$$\tau\frac{d\mathbf{P}}{dt} = -\mathbf{P} + \mathbf{F}_{mig}, \tag{S9}$$

$$\mathbf{p}(t) = \mathbf{P}(t)/|\mathbf{P}(t)|, \tag{S10}$$

where $\mathbf{P}$ represents a vector that temporarily memorizes $\mathbf{F}_{mig}$ in a history-dependent manner and τ indicates time constant. The direction of the persistent force, $\mathbf{p}$, was simply determined by unit vector of $\mathbf{P}$. Parameter values used in equations (S6, S8 and S9) for the 2D simulation of the growth cone migration were γ=0.005, v=15 (μm/hr) and τ=10 (min).

Evaluation of the turning angle

The turning angle of the 2D growth cone was evaluated at 1.5 hours after application of the gradient as

$$\omega = \frac{360}{2\pi}\mathrm{sgn}\left(\mathbf{q}(0)^{\mathrm{T}}\Delta\mathbf{r_c}(t_{eva})\right)\arccos\left[\mathbf{p}(0)^{\mathrm{T}}\Delta\mathbf{r_c}(t_{eva})\right], \tag{S11}$$

where $\varDelta\mathbf{r_c}(t_{eva})$ and $\mathbf{q}(0)$ represent a unit vector of the growth cone displacement from initial position as $\varDelta\mathbf{r_c}(t_{eva})=\{\mathbf{r_c}(t_{eva})-\mathbf{r_c}(0)\}/|\mathbf{r_c}(t_{eva})-\mathbf{r_c}(0)|$ and a vector rotated by -90° with respect to $\mathbf{p}(0)$, respectively, t_{eva} indicates the time point for evaluating the turning angle (1.5 hours), and sgn(s) is a sign function; 1 if $s \geq 0$ and –1 $s < 0$.

Implementation of 2D model simulation

Simulations were implemented by ourselves using MATLAB (MathWorks). In our implementation, we quantized the 2D space into square lattice, in which the size of each square was set to 0.1 μm, so that $\mathbf{r}_{ij}=(i\Delta x, j\Delta x)^{\mathrm{T}}$ denotes the center position of a compartment indexed by (i, j) $(i, j=1, 2, 3, \ldots)$ and $\Delta x=0.1$ μm. The lattice was fixed and not re-meshed. The morphology of the growth cone was represented by a fixed ellipse. At each time step in simulation, the ellipse-shaped growth cone was relocated by updating its centroid position vector and moving vector, according to equation (S7). After this migration, square compartments *inside* the relocated ellipse were re-considered as the growth cone region, leading to another calculation of its centroid and moving vectors.

A square compartment indexed by (i, j) received the level of guidance signal (G) as $G_{ij}=G(\mathbf{r}_{ij})$, where $G(\mathbf{r})$ was given by equation (S5) representing the external gradient (**Supplementary figure 1A**). The levels of activator (A) and inhibitor (I) were determined by $A_{ij}=A(G_{ij})$ and $I_{ij}=I(G_{ij})$, where $A(G)$ and $I(G)$ are dose-response functions of A and I to G, respectively. The level of effector X (X) for each component was determined by a ratio of A to I as $X_{ij}= A_{ij}/I_{ij}$. The set of A_{ij}. I_{ij}, and X_{ij} over the intracellular compartments in total represent the intracellular gradients (as two-dimensional distributions) of A, I, and X, respectively (**Supplementary figure 1C and D**).

Comparison of our theory based on 1D model with 2D model simulation

Because our theory was based on linear approximation (first-order Taylor expansion) in the 1D model, we evaluated performance of our theory compared with simulation of the 2D model. Under a 10% gradient typically used in the growth cone turning assays[4,5] (**Supplementary figure 1A and B**), we showed that our theory well reproduced the simulated turning angle in the 2D model (**Supplementary figure 2**). In addition, we compared our theory with the 2D model simulation by changing the steepness of the extracellular gradient (5, 10, 20 and 30%) (**Supplementary figure 3**). Although approximation of our theory got worse as the gradient steepness increased, we found our 1D model-based theory well reproduced the 2D model simulation reasonably with a gradient range of 5-10%. Therefore, our theory based on the 1D model well characterized the 2D migration of the growth cone in a biologically realistic range of gradients, consistently with the values used in the growth cone turning assay[4,5], but was not very effective for further steeper gradients.

Calculation of ΔA and ΔI

We evaluated ΔA and ΔI, which were used in equation (7) in the main text, as results of intracellular reaction-diffusion dynamics of activator and inhibitor. The reaction-diffusion dynamics of A and I under exposure to a gradient of guidance signal, $G(x)$, are generally described by

$$\frac{\partial Z}{\partial t} = D\frac{\partial^2 Z}{\partial x^2} + h(Z) + wG(x), \quad \text{(S12)}$$

where Z is the concentration of either A or I, D and w indicate diffusion constant and influx rate of Z regulated by the guidance signal, and $h(Z)$ represents a reaction term. When the extracellular gradient is shallow relative to the size of the cell, it can be represented as a linear gradient as $G(x)=G^*+gx$, where G^* and g indicate the basal level and slope of the guidance signal. Then, equation (S12) can be rewritten by

$$\frac{\partial (Z^*+z)}{\partial t} = D\frac{\partial^2 (Z^*+z)}{\partial x^2} + h(Z^*+z) + w\left(G^*+gx\right), \quad \text{(S13)}$$

where $Z=Z^*+z$, Z^* indicates a steady state response of Z in equation (S12) given G^*, i.e., $h(Z^*)+wG^*=0$. Notice that Z^* is just a constant, so $\partial Z^*/\partial t=0$, and that z represents perturbation from Z^*, which took a small value due to the shallow gradient assumption, leading to the linearization of $h(Z^*+z)$ as $h(Z_o+z) \simeq h(Z_o) + dh/dZ\big|_{Z=Z^*} z$. Then, equation (S13) becomes

$$\frac{\partial (Z^*+z)}{\partial t} \simeq D\frac{\partial^2 (Z^*+z)}{\partial x^2} + h(Z^*) + \left.\frac{dh}{dZ}\right|_{Z=Z^*} z + w\left(G^*+gx\right). \quad \text{(S14)}$$

Applying $h(Z^*)+wG^*=0$, we obtain

$$\frac{\partial z}{\partial t} = D\frac{\partial^2 z}{\partial x^2} - kz + wgx, \quad \text{(S15)}$$

where

$$k = -\left.\frac{dh}{dZ}\right|_{Z=Z^*}. \quad \text{(S16)}$$

To easily obtain the solution of equation (S15), we approximately used a model of two compartments, described by

$$\begin{aligned} \frac{dz_r}{dt} &= -D\frac{z_r - z_l}{L^2} - kz_r + \frac{wg}{2}, \\ \frac{dz_l}{dt} &= D\frac{z_r - z_l}{L^2} - kz_l - \frac{wg}{2} \end{aligned} \quad \text{(S17)}$$

where z_r and z_l indicate the z values at right and left compartments, respectively. Difference between z_r and z_l follows

$$\frac{d(\Delta Z)}{dt} = -\left(\frac{2D}{L^2} + k\right)\Delta Z + wg, \quad \text{(S18)}$$

where $\Delta Z=z_r-z_l$. Then, spatial difference of Z at steady state becomes

$$\Delta Z = \frac{wg}{k + 2D/L^2}. \quad \text{(S19)}$$

If $2D/L^2 << k$, this equation is approximately expressed as

$$\Delta Z \simeq g\left(1-\frac{2D}{kL^2}\right)\frac{w}{k}. \qquad \text{(S20)}$$

Because w/k in equation (S20) is shown to correspond to the derivative of the dose-response (see below), ΔZ is also expressed by

$$\Delta Z \simeq c_Z \frac{dF_Z(G)}{dG}, \qquad \text{(S21)}$$

where $F_Z(G)$ indicates the dose-response of Z depending on G, c_z indicates a positive constant that corresponds to the Z's sensitivity to the extracellular gradient, depending on its diffusion constant. This result was used in equation (7) in the main text.

Here, we show $w/k=dF_Z/dG$. In general, the dose-response describes a steady state response of output variable given input, which is also regardless of spatial factor. Thus, we consider the steady state condition of equation (S12) excluding the diffusion term, or, after the diffusion has vanished, leading to $h(Z)+wG=0$. The dose response function is simply given as a solution of this equilibrium equation, as, $Z=F_Z(G)$. Differentiation of $h(F_Z(G))+wG=0$ with respect to G, using the chain rule, $(dh/dZ_{Z=Fz(G)})(dF_Z/dG)+w=0$, leads to the derivative of the dose-response, $dF_Z/dG=-w/(dh/dZ_{Z=Fz(G)})$. Because of equation (S16), $dF_Z/dG=w/k$. Notice that Z^* in equation (S16) satisfies $Z^*=F_Z(G^*)$.

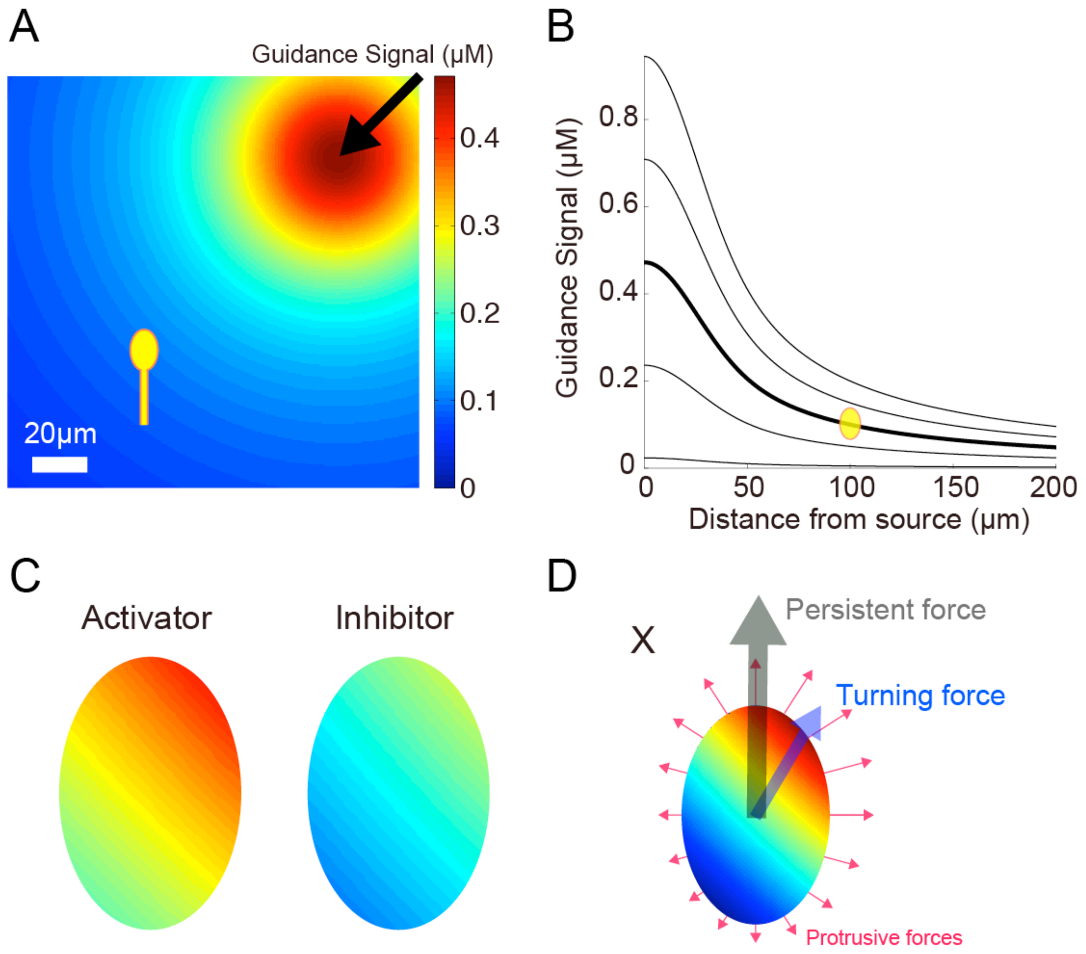

Supplementary Figure 1 Two-dimensional model of the growth cone

(A) The 2D growth cone is depicted by a yellow ellipse-shaped object with 10 μm short and 15 μm long axes. The heat map represents a gradient typically used in the turning assay, in which chemical solution was applied as the black arrow, at 100 μm from the center and 45° from the initial migrating direction of the growth cone.

(B) Concentration of the guidance signal along the diagonal line from the top-right corner in (A). The thick black line corresponds to the situation in (A) and thin black lines represent the situations when variety of concentration of the chemical source was applied. The yellow ellipse is depicted to see the width of the growth cone in (A) with respect to the gradient slope; 10.45% concentration difference between the near and far sides of the growth cone.

(C) Scheme of spatial distributions of activator and inhibitor within the 2D model growth cone in response to the gradient in (A). The scale of the ellipse-shaped growth cone was the same as in (A).

(D) Scheme of turning driven by spatial distribution of X in the 2D model growth cone. The motion of the growth cone was driven by two kinds of forces: persistent and turning forces. The persistent force (black arrow) was constitutively generated even without the extracellular gradient, whereas the turning force (blue arrow) was regulated by polarized distribution of X. Protrusive forces (red arrows) were locally generated along the corresponding normal vector and in proportion to the local activity of X, so that the integration of all protrusive forces became the turning force (blue arrow). The scale of the ellipse-shaped growth cone was the same as in (A).

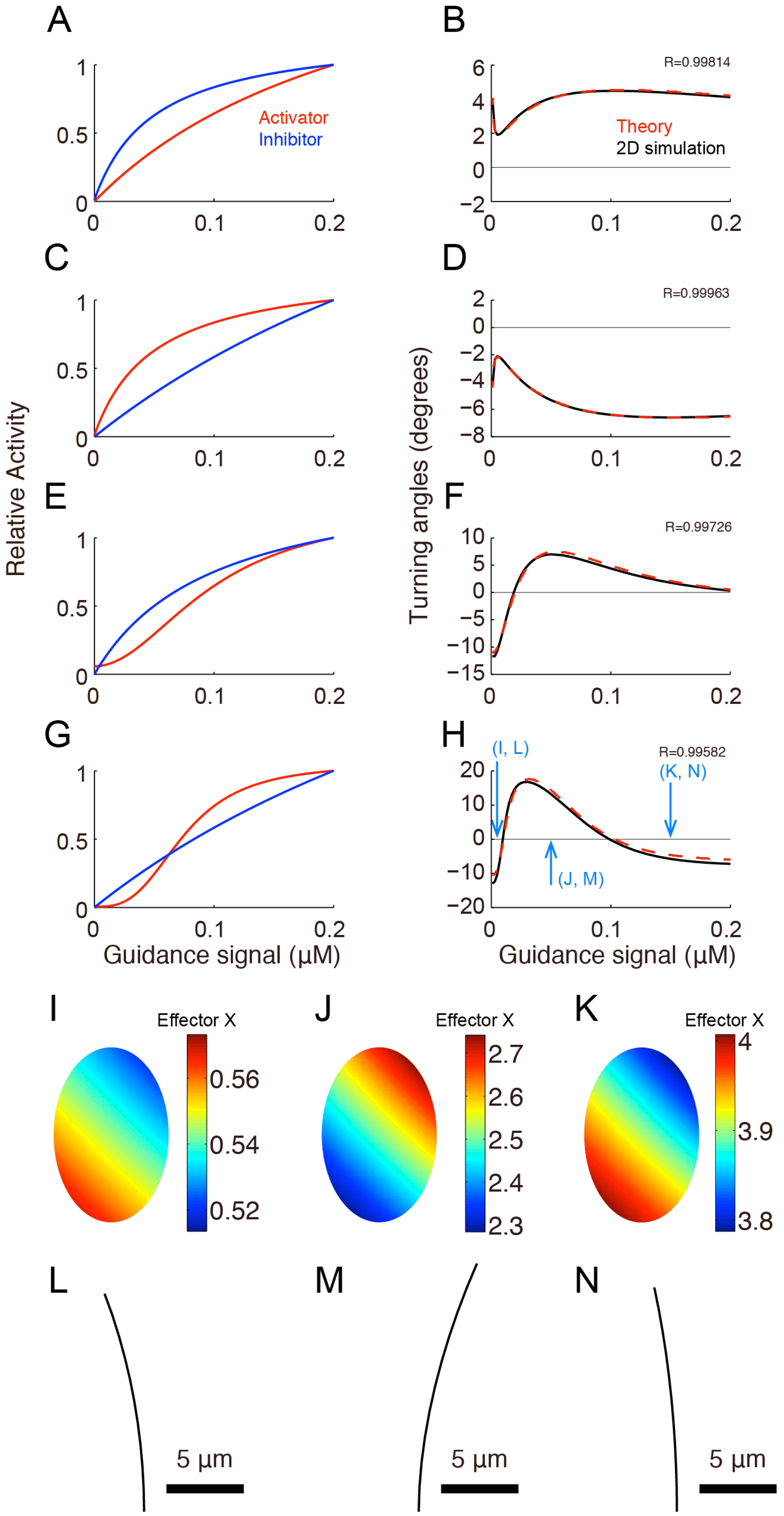

Supplementary Figure 2. Comparison between theory based on 1D model and simulation of 2D model.

(A, C, E and G) The same as Figure 2.

(B, D, F and H) Black lines indicate the simulated turning angles in the 2D model in response to the extracellular gradient shown in supplementary figure 1A. Red dotted lines indicate the turning angles calculated by equation (7), where the amplitude (β) was tuned to fit the black lines.

(I-K) Simulated distributions of X within the growth cone, when the growth cone initially encountered the gradient of guidance cue. Each indexed panel corresponds to a single blue arrow with the same index in (H).

(L-N) Simulated paths of the 2D growth cone model. Each indexed panel corresponds to a single blue arrow with the same index in (H).

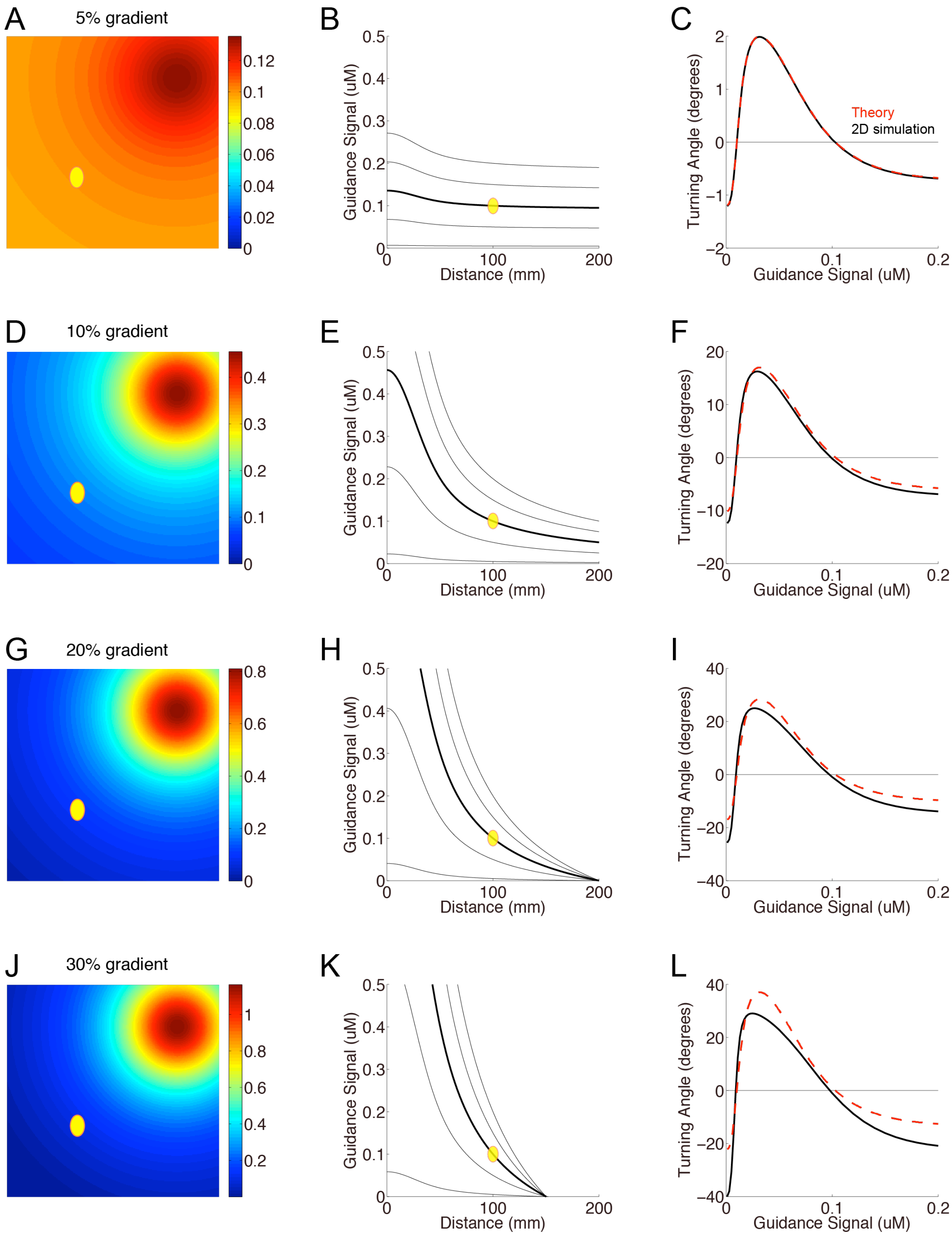

Supplementary Figure 3 Comparison between our theory based on 1D model and 2D model simulation with changing steepness of the extracellular gradients

Various gradients were applied; 5% (A-C), 10% (D-F), 20% (G-I), 30% (J-L) concentration difference between the near and far sides of the growth cone.

(A, D, G, J) The same as supplementary figure 1A.

(B, E, H, K) The same as supplementary figure 1B.

(C, F, I, L) Black lines indicate the turning angles simulated by the 2D model with the dose-responses of A and I shown in Figure 2G. Red dotted lines indicate the turning angles calculated by equation (7), in which the amplitude (β) was tuned to fit the black lines.

References

1 Ferrell, J. E., Jr. Tripping the switch fantastic: how a protein kinase cascade can convert graded inputs into switch-like outputs. *Trends in biochemical sciences* **21**, 460-466 (1996).

2 Goldbeter, A. & Koshland, D. E., Jr. An amplified sensitivity arising from covalent modification in biological systems. *Proceedings of the National Academy of Sciences of the United States of America* **78**, 6840-6844 (1981).

3 Roccasalvo, I. M., Micera, S. & Sergi, P. N. A hybrid computational model to predict chemotactic guidance of growth cones. *Sci Rep* **5**, 11340, doi:10.1038/srep11340 (2015).

4 Lohof, A. M., Quillan, M., Dan, Y. & Poo, M. M. Asymmetric modulation of cytosolic cAMP activity induces growth cone turning. *J Neurosci* **12**, 1253-1261 (1992).

5 Zheng, J. Q., Felder, M., Connor, J. A. & Poo, M. M. Turning of nerve growth cones induced by neurotransmitters. *Nature* **368**, 140-144, doi:10.1038/368140a0 (1994).

6 Keller, E. F. & Segel, L. A. Traveling bands of chemotactic bacteria: a theoretical analysis. *Journal of theoretical biology* **30**, 235-248 (1971).
